# North American Big Brown Bats (Eptesicus fuscus) Harbor an Exogenous *Deltaretrovirus*

**DOI:** 10.1128/mSphere.00902-20

**Published:** 2020-09-23

**Authors:** Ben M. Hause, Eric A. Nelson, Jane Christopher-Hennings

**Affiliations:** a Animal Disease Research and Diagnostic Laboratory, South Dakota State University, Brookings, South Dakota, USA; b Department of Veterinary and Biomedical Sciences, South Dakota State University, Brookings, South Dakota, USA; University of Pittsburgh

**Keywords:** bat, emerging virus, public health, retrovirus, zoonosis

## Abstract

Bats are the reservoir for a large number of zoonotic viruses, including members of *Coronaviridae* (severe acute respiratory syndrome coronavirus [SARS-CoV] and SARS-CoV-2), *Paramyxoviridae* (Hendra and Nipah viruses), *Rhabdoviridae* (rabies virus), and *Filoviridae* (Ebola virus) as exemplars. Many retroviruses, such as human immunodeficiency virus, are similarly zoonotic; however, only infectious exogenous gammaretroviruses have recently been identified in bats. Here, viral metagenomic sequencing of samples from bats submitted for rabies virus testing, largely due to human exposure, identified a novel, highly divergent exogenous *Deltaretrovirus* from a big brown bat (Eptesicus fuscus) in South Dakota. The virus sequence, corresponding to Eptesicus fuscus deltaretrovirus (EfDRV), comprised a nearly complete coding region comprised of canonical 5′-*gag*-*pro*-*pol*-*env*-3′ genes with 37% to 51% identity to human T-lymphotropic virus (HTLV), an infectious retrovirus that causes T-cell lymphoma. A putative *tax* gene with 27% identity to HTLV was located downstream of the *pol* gene along with a gene harbored in an alternative reading frame which possessed a conserved domain for an Epstein-Barr virus nuclear antigen involved in gene transactivation, suggesting a regulatory function similar to that of the deltaretrovirus *rex* gene. A TaqMan reverse transcriptase PCR (RT-PCR) targeting the *pol* gene identified 4/60 (6.7%) bats as positive for EfDRV, which, combined with a search of the E. fuscus genome that failed to identify sequences with homology to EfDRV, suggests that EfDRV is an infectious exogenous virus. As all known members of *Deltaretrovirus* can cause malignancies and E. fuscus is widely distributed in the Americas, often with a colonial roosting behavior in human dwellings, further studies are needed to investigate potential zoonosis.

**IMPORTANCE** Bats host a large numbers of viruses, many of which are zoonotic. In the United States, the big brown bat (Eptesicus fuscus) is widely distributed and lives in small colonies that roost in cavities, often in human dwellings, leading to frequent human interaction. Viral metagenomic sequencing of samples from an E. fuscus bat submitted for rabies testing identified the first exogenous bat *Deltaretrovirus*. The E. fuscus deltaretrovirus (EfDRV) genome consists of the typical deltaretrovial 5′-*gag*-*pro*-*pol*-*env*-3′ genes along with genes encoding two putative transcriptional transactivator proteins distantly related to the Tax protein of human T-cell lymphotrophic virus and nuclear antigen 3B of Epstein-Barr virus. Searches of the E. fuscus genome sequence failed to identify endogenous EfDRV. RT-PCR targeting the EfDRV *pol* gene identified 4/60 (6.7%) bats with positive results. Together, these results suggest that EfDRV is exogenous. As all members of *Deltaretrovirus* are associated with T- and B-cell malignancies or neurologic disease, further studies on possible zoonosis are warranted.

## Abstract (precis)

Bats host a large numbers of viruses, many of which are zoonotic. In the United States, the big brown bat (Eptesicus fuscus) is widely distributed and lives in small colonies that roost in cavities, often in human dwellings, leading to frequent human interaction. Viral metagenomic sequencing of samples from an E. fuscus bat submitted for rabies testing identified the first exogenous bat *Deltaretrovirus*. The E. fuscus deltaretrovirus (EfDRV) genome consists of the typical deltaretrovial 5′-*gag*-*pro*-*pol*-*env*-3′ genes along with genes encoding two putative transcriptional transactivator proteins distantly related to the Tax protein of human T-cell lymphotrophic virus and nuclear antigen 3B of Epstein-Barr virus. Searches of the E. fuscus genome sequence failed to identify endogenous EfDRV. RT-PCR targeting the EfDRV *pol* gene identified 4/60 (6.7%) bats with positive results. Together, these results suggest that EfDRV is exogenous. As all members of *Deltaretrovirus* are associated with T- and B-cell malignancies or neurologic disease, further studies on possible zoonosis are warranted.

## OBSERVATION

Bats are a large, ancient, and incredibly diverse order of mammals that inhabit all continents and ecosystems other than Antarctica (1). Importantly, bats are the reservoir for a large number of viruses, many of which are zoonotic (2). Recent significant human epidemics resulting from zoonotic transmission from bats include those caused by severe acute respiratory syndrome coronavirus, henipaviruses (Hedra and Nipah viruses), Menangle virus, and Ebola virus and, likely most recently, the global pandemic caused by severe acute respiratory syndrome coronavirus 2 (3). Additionally, bats are a common reservoir for rabies virus.

The family *Retroviridae* consists of a large, diverse array of single-stranded RNA (ssRNA) viruses that cause a number of clinically and economically significant diseases (4). A distinguishing feature of the retrovirus life cycle is the formation of a linear double-stranded DNA (dsDNA) genome that can integrate into the host genome to form a provirus. This integration into the host genome can disrupt key cell cycle pathways, leading to malignant transformations and disease such as cancer, immunodeficiencies, autoimmune disorders, or inflammatory diseases. Retroviruses can spread horizontally as infectious agents referred to as exogenous or vertically once integrated into the genome as a provirus. Remnant or “fossil” retroviruses are common in vertebrate genomes and comprise significant amounts of the host genome (5, 6).

Compared to other *Retroviridae* genera, *Deltaretrovirus* consists of only a small number of viruses with limited host range (4). There are three recognized species of primate T-lymphotrophic virus (TLV) that infect humans (HTLV) and simians (STLV). While the persistent infection is often asymptomatic, 4% to 5% of HTLV-1 infections progress to leukemia or lymphoma (7). HTLV-1 can also cause HTLV-1-associated myelopathy/tropical spastic paraparesis (8).

The remaining *Deltaretrovirus* species, bovine leukemia virus (BLV), causes losses to the U.S. dairy industry in excess of 200 million dollars annually due to decreased cow longevity, milk production, and sale price due to carcass condemnation resulting from lymphoma (9). Additionally, BLV has an immunosuppressive effect on the bovine immune system (10). BLV has been eradicated from many countries, so it also represents a trade barrier. More troubling are multiple studies that have associated the detection of BLV in human breast tissue with breast cancer (11).

Compared to domestic animals, very little viral surveillance is performed in wildlife. Bats are a reservoir of rabies virus and represent the most commonly detected rabies carriers in the United States. Additionally, bats are the reservoir of numerous zoonotic diseases worldwide and so are of particular concern when exposed to humans (1, 12). Here, we utilized big brown bats (Eptesicus fuscus) submitted for rabies detection that tested negative for rabies virus to screen for potential novel and zoonotic viruses using viral metagenomic sequencing.

All of the bats (*n* = 60) were submitted to the South Dakota State University Animal Disease Research and Diagnostic Laboratory between March and July 2020. The lungs and heart were dissected from 20 bats and homogenized in a minimal volume of phosphate-buffered saline before centrifugation was performed at 20,000 × *g* for 5 min to pellet debris. Next, 80 μl of sample was transferred to a microcentrifuge tube and treated with a cocktail of nucleases to digest unprotected nucleic acids. Nucleic acid isolation, reverse transcription, second strand synthesis, and amplification were performed as previously described (13). Sequencing libraries were constructed for each bat individually using a Nextera XT library preparation kit followed by sequencing on a MiSeq instrument using paired 151-bp reads. Approximately 200,000 to 500,000 reads were generated per sample. Sequenced reads were trimmed of adapter sequences using onboard software before being exported to CLC Genomics and assembled *de novo*. Contig sequences were analyzed by BLASTX using the BLAST2Go plugin incorporated into the software package.

One sample contained three contigs (691 to 1,259 bp) most similar to HTLV. Metagenomic sequencing was performed on this sample multiple times. Approximately 1.1 million combined sequencing reads were used to assemble a contig 6,597 bp in length that was verified by Sanger sequencing. The sample was collected from a bat found dead in a private residence in South Dakota.

**FIG 1:**
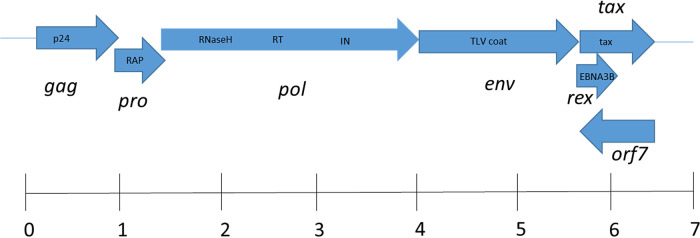
Genomic structure of Eptesicus fuscus deltaretrovirus. Open reading frames (ORF) corresponding to *gag*, *pro*, *pol*, *env*, and *tax* were identified by BLASTP based on homology with primate T-cell lymphotropic virus. The ORF harboring the putative *rex* gene contains an Epstein-Barr virus nuclear antigen 3B (EBNA3B) conserved domain. Conserved retroviral domains identified by BLASTP are shown in boxes within each ORF. ORF7 had no similarity to known proteins or conserved domains. Abbreviations: retroviral aspartyl protease (RAP), reverse transcriptase (RT), integrase (IN). The scale bar indicates size in kilobases.

Open reading frame (ORF) and BLASTP analysis identified viral proteins Gag-Pro-Pol-Env with 37% to 51% identity to proteins encoded by HTLV and STLV (Fig. 1). Translation of the overlapping Gag, Pro, and Pol proteins presumably occurs due to ribosomal −1 frameshifts, as a presumptive slippage sequence, 6(A)-9 nt (nucleotides)-5(G)-10 nt-5(C), was identified at the 3′ end of *gag* in the *pro* overlap region that is very similar to the conserved slippage sequence 6(A)-8 nt-6(G)-11 nt-6(C). Similarly, a ribosomal slippage sequence matching HTLV-1 and HTLV-2 (TTTAAAC) was identified near the predicted 3′ end of *pro* in the region that overlaps *pol*. A putative Tax homolog was identified immediately downstream of *env* based on 25% identity with HTLV. An ORF-overlapping *env* encoded a predicted 238-amino-acid (a.a.) protein which failed to show significant homology to known proteins by BLASTP; however, it did possess a conserved domain for an Epstein-Barr virus nuclear antigen involved in gene transactivation, suggesting a regulatory role in viral replication similar to that identified for the deltaretrovirus *rex* gene (14). A final ORF encoding a 316-a.a. protein was identified on the complementary strand that overlaps the putative *tax* and *rex* ORFs. This predicted protein showed no similarity to known proteins by BLASTP analysis. Similarly to other deltaretroviruses, the nucleotide composition strongly favored cytosine (32.4%) compared to adenine, guanine, and thymine (21.0% to 24.4%).

**FIG 2:**
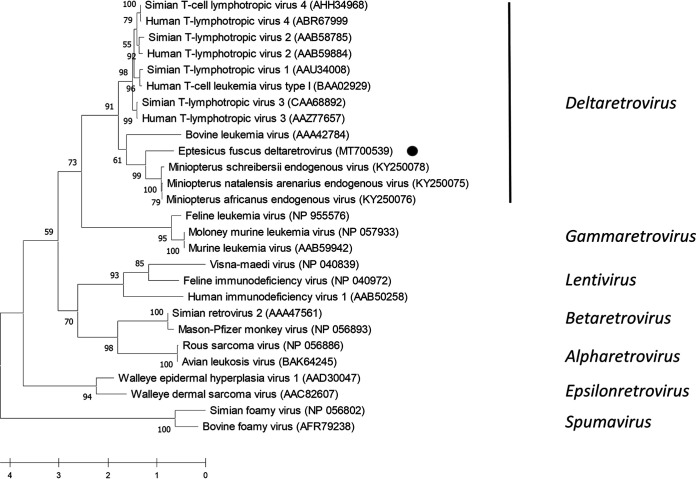
Phylogenetic analysis of the partial Gag protein (374 amino acids). Sequences were aligned by the use of ClustalW, and the phylogenetic tree was inferred by maximum-likelihood analysis using the Whelan and Goldman +F model implemented in MegaX (16). The robustness of the tree topology was evaluated with 1,000 bootstrap replicates. The scale bar indicates number of amino acid substitutions per site. Positions with less than 80% coverage were eliminated from the analysis. Eptesicus fuscus deltaretrovirus (EfDRV) is indicated by a bullet symbol (•).

Phylogenetic analysis was performed on partial Gag amino acid sequences using representatives from all *Retroviridae* genera. Also included in this analysis were Gag sequences from an endogenous *Deltaretrovirus* identified in *Miniopteridae* genomes (15). Sequences were aligned in MegaX by the use of ClustalW followed by phylogenetic reconstruction using the best-fitting Whelan and Goldman frequency model (16). Tree topology was evaluated with 1,000 bootstrap replicates. EfDRV formed a sister group to endogenous *Miniopteridae* deltaretroviruses in a clade with BLV in the ancestral position (Fig. 2). A sister clade contained primate T-lymphotrophic viruses. Similar analyses performed with the Pol and Env amino acid sequences placed EfDRV in the same taxonomic position (data not shown). Previous work found that deltaretrovirus endogenization in *Miniopteridae* occurred 20 to 45 million years ago, suggesting that EfDRV has been circulating in bats for at least that amount of time (15). More recently, endogenous deltaretrovirus sequences were identified in cetaceans, insectivores, and carnivores (17).

A BLASTN search of the Eptesicus fuscus genome failed to find any significant matches (expectation values ≥ 0.03) to the EfDRV genome. Next, a TaqMan PCR was designed targeting the EfDRV *pol* gene using the following primers and probe: forward primer, 5′-CTATTTCCTGGCTACTGACACC; reverse primer, 5′-GGTAAGGTAGTGATGGTGCG; probe, 5′-6-carboxyfluorescein (FAM)-CCTCGTGATTTGGCTCCTTGGGT. Quantitative reverse transcriptase PCR (QRT-PCR) was performed on the 20 bat samples subjected to metagenomic sequencing and additionally on 40 viscera homogenates prepared from bats submitted for rabies virus testing. Four of the 60 (6.7%) bats were positive for EfDRV (threshold cycle [*C_T_*] values, 26.5 to 33.5), including the sample where EfDRV was identified by metagenomic sequencing. Amplification and sequencing of an 847-bp product spanning parts of the *pro* and *pol* genes using primers 621F (5′-CTCCTGCGAGCTTGTGCTAATG) and 1624R (5′-GGGAGAACTTCCCAAGCGTAAC) found 97% to 100% identity to the EfDRV genome. Treatment of sample nucleic acids with DNase did not affect Eptesicus fuscus deltaretrovirus (EfDRV) detection by QRT-PCR, while treatment with RNase rendered EfDRV undetectable. Together, these results suggest that EfDRV is an exogenous infectious virus.

Recently, multiple exogenous gammaretroviruses related to koala retrovirus were identified in bats from Asia and Australia (18). Prior to this report, only endogenous retroviruses originating from multiple genera had been identified in bat genomes (15, 19, 20). The infection of koalas with koala retrovirus occurs both vertically and horizontally (21, 22). Koala retrovirus has been endogenized in Northern Australia koalas, while animals in the south are infected horizontally. Infection with koala retrovirus has been associated with the development of lymphoma and immunosuppression linked to *Chlamydia* infection (21, 23–25). Interestingly, EfDRV shows a well-supported evolutionary relationship to *Miniopterus* endogenous retrovirus.

EfDRV is the first described bat *Deltaretrovirus* and represents only the second report of an exogenous bat retrovirus. The identification of EfDRV from a bat with human exposure raises questions of possible zoonosis, considering that all members of this genus cause disease. Further research and surveillance are needed to determine if EfDRV poses a risk to human or animal health.

### Data availability.

The sequence described in this work was submitted to GenBank under accession no. MT700539. Sequence reads were archived as BioProject PRJNA659740.
